# Can Mobile Phone Data Improve Emergency Response to Natural Disasters?

**DOI:** 10.1371/journal.pmed.1001085

**Published:** 2011-08-30

**Authors:** Peter W. Gething, Andrew J. Tatem

**Affiliations:** 1 Spatial Ecology and Epidemiology Group, Department of Zoology, University of Oxford, Oxford, United Kingdom; 2 Emerging Pathogens Institute, University of Florida, Gainesville, Florida, United States of America; 3 Department of Geography, University of Florida, Gainesville, Florida, United States of America; 4 Fogarty International Center, National Institutes of Health, Bethesda, Maryland, United States of America

## Abstract

Peter Gething and Andrew Tatem discuss the potential impact of mobile phone positioning data on disaster response and highlight challenges that must be addressed if use of this technology is to develop.

**Linked Research Article**

This Perspective discusses the following new study published in *PLoS Medicine*:

Bengtsson L, Lu X, Thorson A, Garfield R, von Schreeb J (2011) Improved Response to Disasters and Outbreaks by Tracking Population Movements with Mobile Phone Network Data: A Post-Earthquake Geospatial Study in Haiti. PLoS Med 8(8): e1001083. doi:10.1371/journal.pmed.1001083

Linus Bengtsson and colleagues examine the use of mobile phone positioning data to monitor population movements during disasters and outbreaks, finding that reports on population movements can be generated within twelve hours of receiving data.

Disaster management requires accurate information and must link data collection and analysis to an immediate decision-making process. Existing approaches to assessing population movements in the immediate aftermath of disasters, such as transport surveys and manual registration of individuals at emergency-relief hubs, are often inadequate: while important for record-keeping purposes, both are slow and may exclude those groups who are unreachable and most vulnerable. Proxy analysis via aerial or even satellite reconnaissance has a potentially useful role, but can provide only a coarse geographical picture of moving populations. In practice, the most readily available sources of information are from eye-witness or media reports. Although timely, such reports are not accumulated systematically and can constitute a biased representation of events.

## Mobile Phone Data Can Describe Population Movements

In recent years, awareness has grown of a potentially revolutionary way of tracking movement and mobility of human populations by exploiting data from mobile phone networks. Calls or text messages sent from mobile phones are routed via the nearest network mast. If records of calls made by individual phones are linked to data on mast locations, then the approximate whereabouts of potentially very large numbers of mobile phones and, by proxy, the users of those phones, can be determined. In turn, population movement can be detected by identifying records for which calls from the same phone are routed via different masts over a period of time. The approach is in its infancy but proof-of-principle studies are emerging that demonstrate its value in aiding understanding of human movement patterns [1],[2], inference of social network structures [3], and estimating malaria importation rates [4]. To date, however, the potential of mobile phone data to support population tracking in the chaotic aftermath of a major natural disaster has not been explored. In a new study published by Bengtsson and colleagues in *PLoS Medicine* this week [5], the authors present an analysis of the efforts undertaken to use mobile phone data to estimate the major population displacement that followed the catastrophic Haitian earthquake of 12 January 2010.

## Tracking Population Displacement after the 2010 Haiti Earthquake

Bengtsson and colleagues retrospectively obtained data for a period spanning six weeks before the disaster to five months after, including data from 282 million calls from 2.8 million individual phones. By making some simple assumptions about the ownership of phones, they estimated the numbers of individuals displaced, the timing of major population movements, and the areas of the country to which these individuals travelled. The study team found that their estimates were much closer to the detailed results of a retrospective survey than to the more ad-hoc earlier estimates that were used during the crisis, suggesting that the mobile phone data provide a more detailed and robust picture of population movement than was otherwise available during the disaster response effort.

A crucial question, however, is whether the approach presented by Bengtsson and colleagues can be operationalised, with data obtained and analysed, and results disseminated over the rapid time scales required by response coordinators. The authors addressed this issue in a smaller second phase of the study, applying their approach in close to real-time and tracking the outflow of individuals from a post-earthquake cholera outbreak focus to uninfected parts of the country. They were able to implement their analyses and disseminate results within 12 hours of acquiring the network data.

## Challenges for Phone Data in Disaster Response

The potential advantages of mobile phone data for population tracking are self-evident: data are abundant, timely, and require no dedicated survey or collection effort. However, some important limitations must be addressed if this potential application is to be realised. Many of these are discussed by Bengtsson and colleagues in the context of disaster relief, but apply equally to the wider uses of phone data being developed for public health applications. First, natural disasters themselves can damage mobile phone networks, limiting their coverage, data utility, and data availability, while damage to power grids limit users' ability to recharge their handsets. Moreover, the density of masts is usually tightly correlated with residential population density, meaning that displacements into rural areas, where masts are often hundreds of kilometres apart, are difficult to track accurately. Cross-border displacements are also exceptionally challenging to track because of the national divisions of network operator companies, potentially limiting applications in many emergency situations. Second, the representativeness of mobile phone data remains an important question both in disaster situations and more generally. Mobile phone ownership levels remain uncertain in many countries [6], and are likely biased away from women, the poor, rural, young, and elderly, who may be most adversely affected by natural disasters. Lastly, as applications are developed and become more widespread, protocols must be developed to ensure privacy is safeguarded appropriately, as was done by Bengtsson and colleagues.

Integration with formal disaster response planning will require close cooperation and coordination between different network operators that may control different percentages of the market and maintain call data in a variety of formats. Bengtsson and colleagues have demonstrated a valuable proof-of-concept of the use of phone data in disaster response, but substantial further work will likely be required before operational usage becomes common. While millions continue to be adversely affected by natural disasters, in an increasingly connected world where mobile phone ownership is becoming ubiquitous, these data will likely become a valuable component of the disaster response toolbox. Bengtsson and colleagues have taken the first step towards this full potential being realised.
